# Supplementary material for: The LPS-inducible lncRNA Mirt2 is a negative regulator of inflammation
Source: Nat Commun. 2017 Dec 11;8:2049. doi: 10.1038/s41467-017-02229-1 (PMC5725456; doi:10.1038/s41467-017-02229-1)
Supplement: Supplementary file 1 — Supplementary information [file 41467_2017_2229_MOESM1_ESM.pdf]

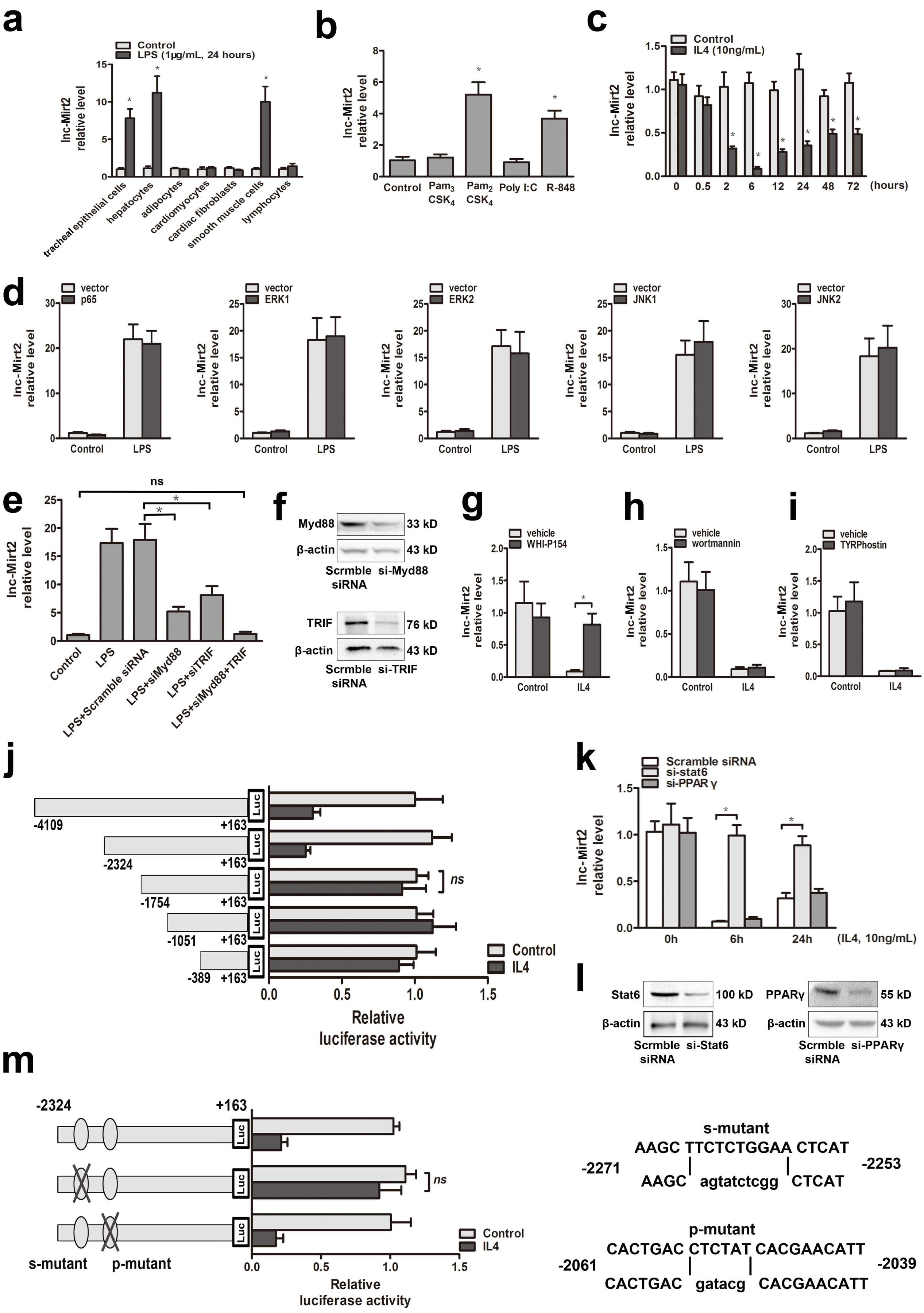
a
Control
LPS (1μg/mL, 24 hours)
lnc-Mirt2 relative level
tracheal epithelial cells
hepatocytes
adipocytes
cardiomyocytes
cardiac fibroblasts
smooth muscle cells
lymphocytes
b
lnc-Mirt2 relative level
Control
Pam3 CSK4
Pam2 CSK4
Poly I:C
R-848
c
Control
IL4 (10ng/mL)
lnc-Mirt2 relative level
0 0.5 2 6 12 24 48 72 (hours)
d
vector
p65
ERK1
ERK2
JNK1
JNK2
lnc-Mirt2 relative level
Control
LPS
e
ns
lnc-Mirt2 relative level
Control
LPS
LPS+Scramble siRNA
LPS+siMyd88
LPS+siTRIF
LPS+siMyd88+TRIF
f
Myd88
33 kD
β-actin
43 kD
Scrmble siRNA
si-Myd88
TRIF
76 kD
β-actin
43 kD
Scrmble siRNA
si-TRIF
g
vehicle
WHI-P154
lnc-Mirt2 relative level
Control
IL4
h
vehicle
wortmannin
lnc-Mirt2 relative level
Control
IL4
i
vehicle
TYRPhostin
lnc-Mirt2 relative level
Control
IL4
j
-4109 +163
-2324 +163
-1754 +163
-1051 +163
-389 +163
Luc
ns
Control
IL4
Relative luciferase activity
k
Scramble siRNA
si-stat6
si-PPAR γ
lnc-Mirt2 relative level
0h
6h
24h
(IL4, 10ng/mL)
l
Stat6
100 kD
β-actin
43 kD
Scrmble siRNA
si-Stat6
PPARγ
55 kD
β-actin
43 kD
Scrmble siRNA
si-PPARγ
m
-2324
+163
Luc
ns
s-mutant
p-mutant
Control
IL4
Relative luciferase activity
s-mutant
AAGC TTCTCTGGAA CTCAT
-2271
-2253
AAGC agtatctcgg CTCAT
p-mutant
CACTGAC CTCTAT CACGAACATT
-2061
-2039
CACTGAC gatacg CACGAACATT

Supplementary Figure 1. IL-4 inhibites Mirt2 expression in macrophages via Jak-Stat6 signaling pathway. (a), The expression of Mirt2 in several kinds of cells treated with LPS (1 μg/mL, 24h). **(b),** The expression of Mirt2 in cultured peritoneal macrophages treated with other TLR ligands, that is, Pam_3CSK_4 (TLR2/1, 300 ng/mL), Pam_2CSK_4 (TLR2/6, 100 ng/mL), Poly (I:C) (TLR3, 25 μg/mL) and R848 (TLR7/8, 10 umol/L). **(c),** The expression of Mirt2 in cultured peritoneal macrophages treated with IL-4 for different times. **(d),** Effects of p65, ERK1, ERK2, JNK1 or JNK2 overexpression on the expression of Mirt2 in macrophages. **(e),** Effects of Myd88 or TRIF knockdown on the expression of Mirt2 in macrophages treated with LPS (1 μg/mL, 24h). **(f),** The knockdown efficiencies for Myd88 and TRIF siRNAs. **(g-i),** Macrophages were pretreated with selective pharmacological inhibitors wortmannin (PI3K), TYRPhostin (Jak2) or WHI-P154 (Jak3), then cells were treated with IL-4. Expression of Mirt2 was detected by qRT-PCR. **(j),** Luciferase reporter constructs containing Mirt2 promoter or its truncations were co-transfected with an internal control plasmid pRL-TK into RAW264.7 cells, followed by IL-4 stimulation. The relative luciferase activities are expressed as a percent of values determined in control group. **(k),** Stat6 or PPARγ in macrophages was knocked down before IL-4 stimulation. Expression of Mirt2 was detected by qRT-PCR. **(l),** The knockdown efficiency for Stat6 siRNA. **(m),** Luciferase reporter constructs containing Mirt2 promoter (-2324bp ～ +163bp) or its mutants (s-mutant for Stat6 binding site; p-mutant for PPARγ binding site) were co-transfected with an internal control plasmid pRL-TK into RAW264.7 cells, followed by IL-4 stimulation. The left panel demonstrates the relative luciferase activities, which are expressed as a percent of values determined in control group. The right panel demonstrates

the mutation for Stat6 and PPARγ binding sites. Data represent the mean ± SEM of three independent experiments. * $P < 0.05$. *ns*, none significant. Two-tailed Student's t-test for two groups and one-way ANOVA for multiple groups.

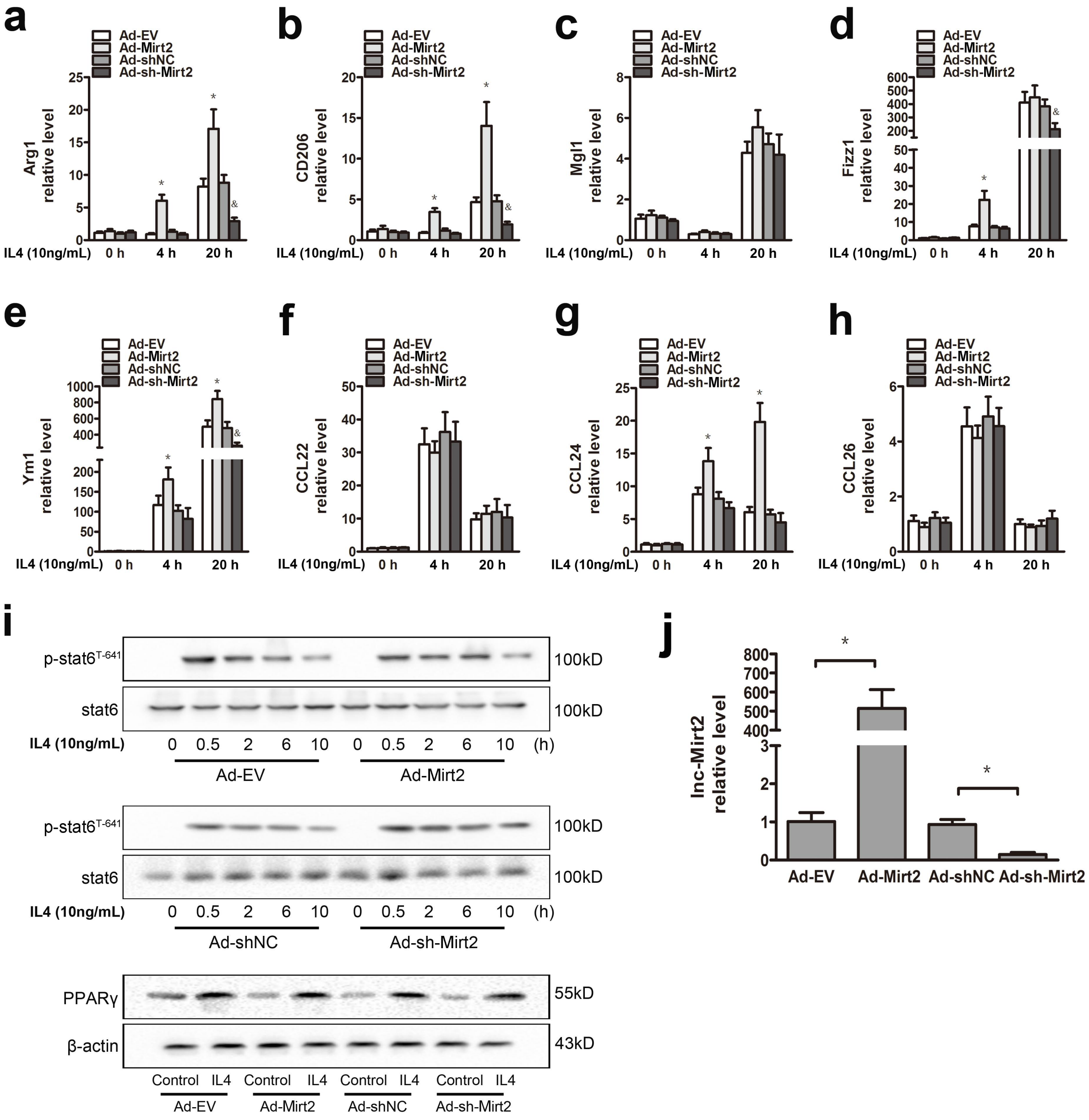

Supplementary Figure 2. Mirt2 promotes IL-4-induced macrophage M2 polarization.

(a-h), Effects of Mirt2 overexpression or Mirt2 knockdown on the expression of M2 markers and cytokines induced by IL-4 in cultured peritoneal macrophages, as determined by

qRT–PCR. Data represent the mean ± SEM of three independent experiments. * $P < 0.05$ vs. Ad-EV or Ad-shNC group. **(i),** Effects of Mirt2 overexpression or Mirt2 knockdown on Stat6 phosphorylation and PPARγ expression induced by IL-4 in cultured peritoneal macrophages, as determined by Western blot. **(j),** The adenovirus infection efficiency was examined by qRT-PCR. One-way ANOVA for multiple groups.

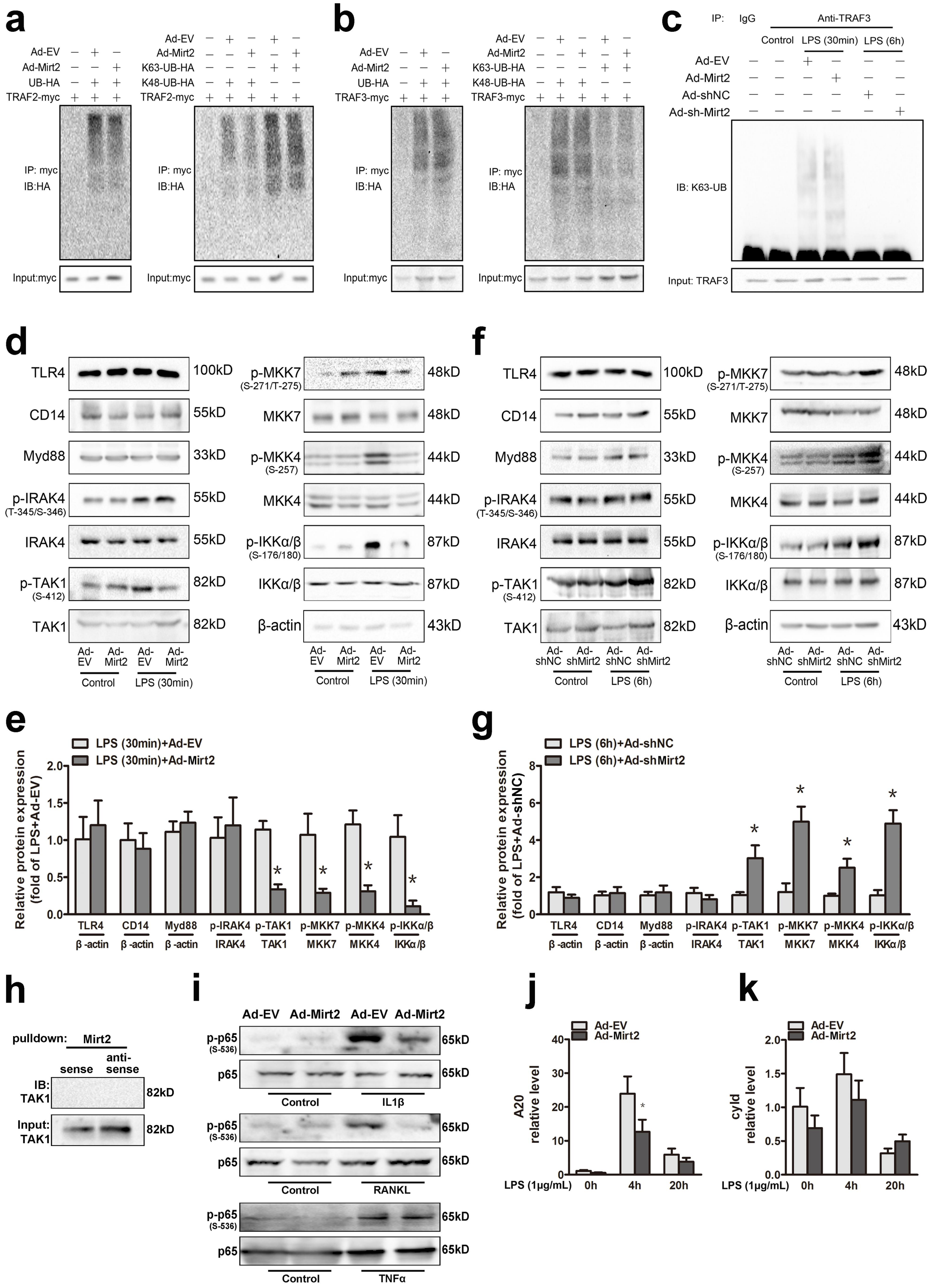
a
Ad-EV
Ad-Mirt2
UB-HA
TRAF2-myc
IP: myc
IB:HA
Input:myc
K63-UB-HA
K48-UB-HA
b
TRAF3-myc
c
IP: IgG
Anti-TRAF3
Control
LPS (30min)
LPS (6h)
Ad-shNC
Ad-sh-Mirt2
IB: K63-UB
Input: TRAF3
d
TLR4
CD14
Myd88
p-IRAK4 (T-345/S-346)
IRAK4
p-TAK1 (S-412)
TAK1
p-MKK7 (S-271/T-275)
MKK7
p-MKK4 (S-257)
MKK4
p-IKKα/β (S-176/180)
IKKα/β
β-actin
100kD
55kD
33kD
82kD
48kD
44kD
87kD
43kD
f
e
LPS (30min)+Ad-EV
LPS (30min)+Ad-Mirt2
Relative protein expression (fold of LPS+Ad-EV)
g
LPS (6h)+Ad-shNC
LPS (6h)+Ad-shMirt2
Relative protein expression (fold of LPS+Ad-shNC)
h
pulldown: Mirt2
sense
anti-sense
IB: TAK1
Input: TAK1
82kD
i
p-p65 (S-536)
p65
65kD
IL1β
RANKL
TNFα
j
A20 relative level
LPS (1μg/mL)
0h
4h
20h
k
cyld relative level

Supplementary Figure 3. Mirt2 has no effects on the auto-ubiquitination of TRAF2 and TRAF3. (a), Immunoblot analysis of the ubiquitination of TRAF2 in HEK293T cells transfected to express myc-tagged TRAF2 and HA-tagged ubiquitin (UB-HA, K48-UB-HA or K63-UB-HA) in the presence or absence of Mirt2, assessed after immunoprecipitation with anti-myc antibody. **(b),** Immunoblot analysis of the ubiquitination of TRAF3 in HEK293T cells transfected to express myc-tagged TRAF3 and HA-tagged ubiquitin (UB-HA, K48-UB-HA or K63-UB-HA) in the presence or absence of Mirt2, assessed after immunoprecipitation with anti-myc antibody. **(c),** Immunoblot analysis of the K63-linked ubiquitination of endogenous TRAF3 in Mirt2-overexpression or Mirt2-knockdown peritoneal macrophages treated with LPS, assessed after immunoprecipitation with TRAF3 antibody. **(d),** Effects of Mirt2 overexpression on the activation of TLR4-related signaling proteins induced by LPS, as determined by Western blot. **(e),** Quantification of band density in panel **d**. **(f),** Effects of Mirt2 knockdown on the activation of TLR4-related signaling proteins induced by LPS, as determined by Western blot. **(g),** Quantification of band density in panel **f**. **(h),** Immunoblotting analysis of proteins in macrophages that are bound to biotinylated Mirt2 or its antisense using anti-TAK1 antibody. **(i),** Effects of Mirt2 on the phosphorylation of p65 induced by IL-1β, RANKL or TNF in macrophages. **(j, k),** Effects of Mirt2 overexpression on the expression of de-ubiquitination enzymes A20 and cyld in macrophages, as determined by qRT–PCR. Data are expressed as mean ± SEM of three independent experiments. * $P < 0.05$ vs. Ad-EV or Ad-shNC group. Two-tailed Student's t-test for two groups.

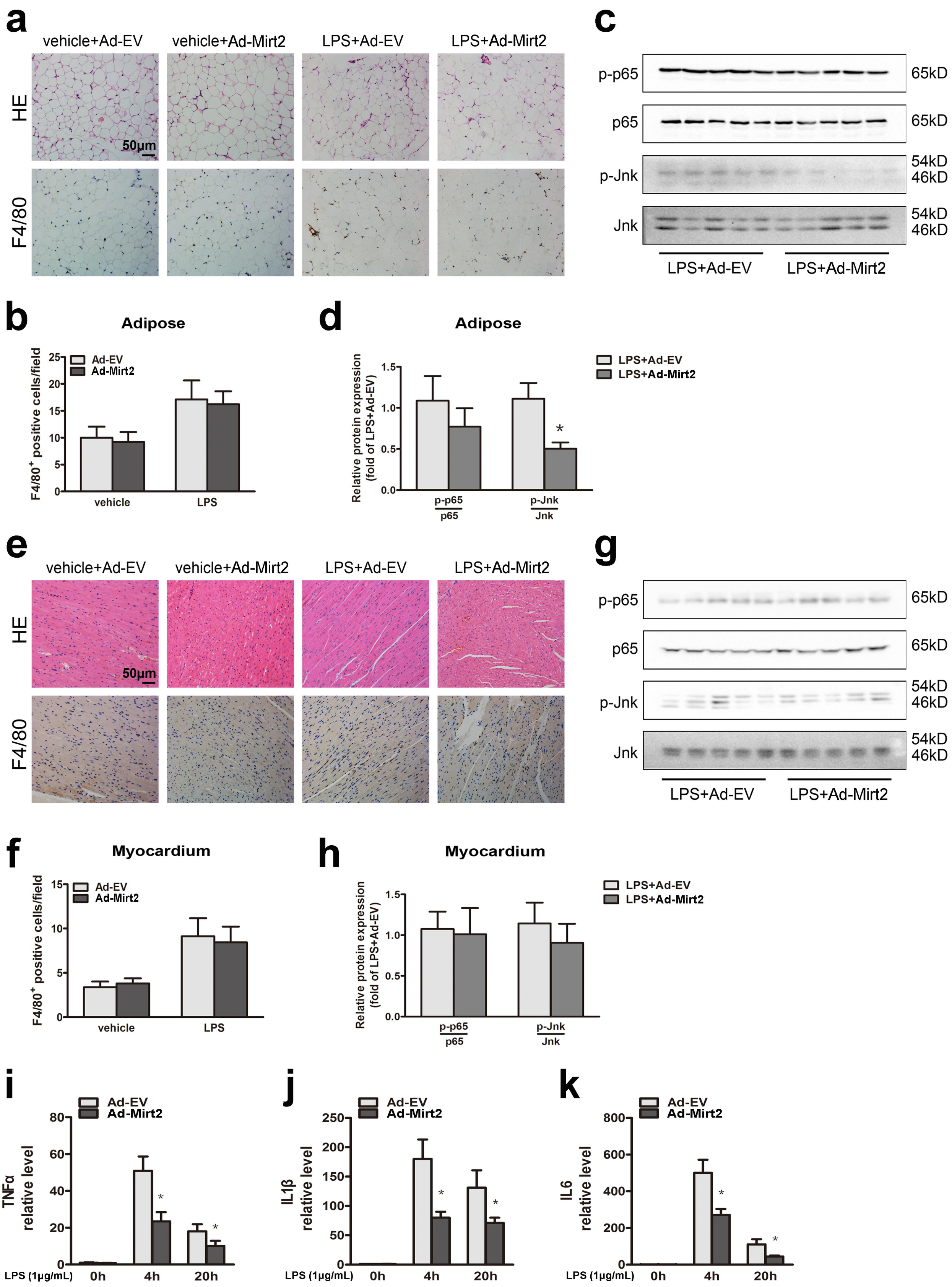
a
vehicle+Ad-EV
vehicle+Ad-Mirt2
LPS+Ad-EV
LPS+Ad-Mirt2
HE
F4/80
50μm
c
p-p65
p65
p-Jnk
Jnk
65kD
65kD
54kD
46kD
54kD
46kD
LPS+Ad-EV
LPS+Ad-Mirt2
b
Adipose
Ad-EV
Ad-Mirt2
F4/80+ positive cells/field
vehicle
LPS
d
Adipose
Relative protein expression (fold of LPS+Ad-EV)
LPS+Ad-EV
LPS+Ad-Mirt2
p-p65/p65
p-Jnk/Jnk
e
vehicle+Ad-EV
vehicle+Ad-Mirt2
LPS+Ad-EV
LPS+Ad-Mirt2
HE
F4/80
50μm
g
p-p65
p65
p-Jnk
Jnk
65kD
65kD
54kD
46kD
54kD
46kD
LPS+Ad-EV
LPS+Ad-Mirt2
f
Myocardium
Ad-EV
Ad-Mirt2
F4/80+ positive cells/field
vehicle
LPS
h
Myocardium
Relative protein expression (fold of LPS+Ad-EV)
LPS+Ad-EV
LPS+Ad-Mirt2
p-p65/p65
p-Jnk/Jnk
i
Ad-EV
Ad-Mirt2
TNFα relative level
LPS (1μg/mL)
0h
4h
20h
j
Ad-EV
Ad-Mirt2
IL1β relative level
LPS (1μg/mL)
0h
4h
20h
k
Ad-EV
Ad-Mirt2
IL6 relative level
LPS (1μg/mL)
0h
4h
20h

Supplementary Figure 4. The effects of Mirt2 on the inflammatory responses of adipose and myocardium in LPS-induced endotoxemia. Endotoxemia was induced in C57BL/6 mice by intraperitoneal injection of LPS (25 mg/kg), and control animals were administered with equivalent volumes of normal saline. Adenovirus (Ad-Mirt2 or Ad-EV) were delivered into mice by tail veil injection 3 days before LPS challenge. **(a),** Histopathology in adipose of Ad-EV or Ad-Mirt2 treated mice 24 h after LPS challenge. *Upper panel:* Hematoxylin and eosin staining; *Lower panel:* Immunohistochemical staining for macrophage marker F4/80. **(b),** Quantitative analysis of F4/80 positive cells. Data are expressed as mean ± SEM (n=8). **(c),** Western blot analysis for the phosphorylation of p65 and Jnk in adipose of endotoxemia mice treated with Ad-Mirt2 or Ad-EV. **(d),** Quantification of band density in panel **c**. Data are expressed as mean ± SEM (n=5). * $P < 0.05$ vs. Ad-EV group. **(e),** Histopathology in myocardium of Ad-EV or Ad-Mirt2 treated mice 24 h after LPS challenge. *Upper panel:* Hematoxylin and eosin staining; *Lower panel:* Immunohistochemical staining for macrophage marker F4/80. **(f),** Quantitative analysis of F4/80 positive cells. Data are expressed as mean ± SEM (n=8). **(g),** Western blot analysis for the phosphorylation of p65 and Jnk in myocardium of endotoxemia mice treated with Ad-Mirt2 or Ad-EV. **(h),** Quantification of band density in panel **g**. Data are expressed as mean ± SEM (n=5). **(i-k),** Adenovirus (Ad-Mirt2 or Ad-EV) were delivered into mice by tail veil injection. After 3 days, primary peritoneal macrophages were prepared, cultured in vitro and challenged with LPS (1 μg/mL) for 4 and 20 hours. The expression of TNF, IL-1β and IL-6 were determinated by qRT-PCR. Data are expressed as mean ± SEM of three independent experiments. * $P < 0.05$ vs. Ad-EV group. Two-tailed Student's t-test for two groups.

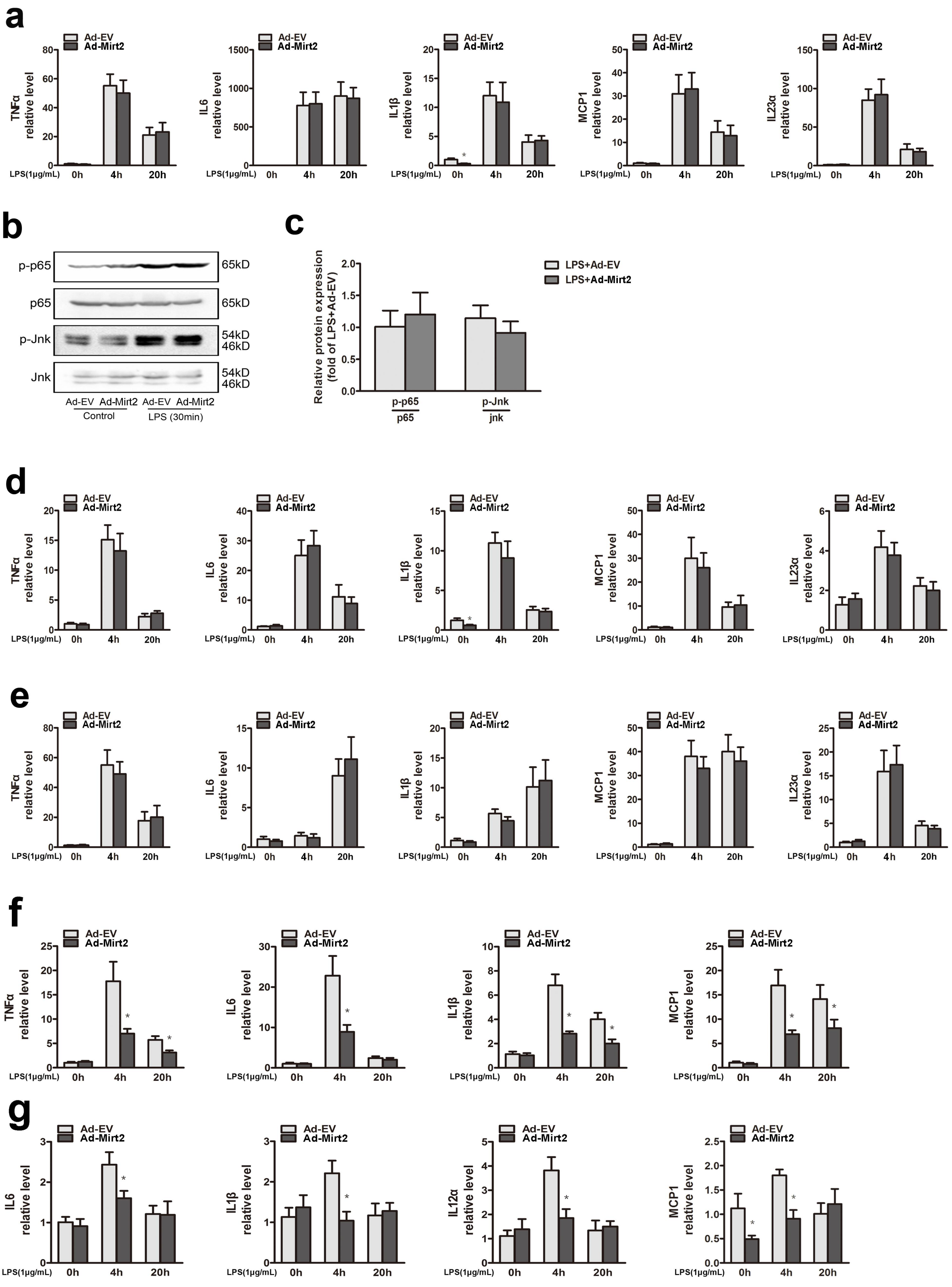
a
Ad-EV
Ad-Mirt2
TNFα relative level
IL6 relative level
IL1β relative level
MCP1 relative level
IL23α relative level
LPS(1μg/mL) 0h 4h 20h
b
p-p65
p65
p-Jnk
Jnk
65kD
54kD
46kD
Ad-EV Ad-Mirt2 Ad-EV Ad-Mirt2
Control
LPS (30min)
c
LPS+Ad-EV
LPS+Ad-Mirt2
Relative protein expression (fold of LPS+Ad-EV)
p-p65 / p65
p-Jnk / jnk
d
e
f
g
IL12α relative level

Supplementary Figure 5. The effects of Mirt2 on the inflammatory responses of adipocytes, cardiomyocytes and cardiac fibroblasts. (a), Effects of Mirt2 on the expression of inflammatory factors in murine adipocytes, as determined by qRT–PCR. **(b),** Effects of Mirt2 on the phosphorylation of p65 and Jnk in murine adipocytes, as determined by Western blot. **(c),** Quantification of band density in panel **b**. **(d),** Effects of Mirt2 on the expression of inflammatory factors in murine cardiomyocytes, as determined by qRT–PCR. **(e),** Effects of Mirt2 on the expression of inflammatory factors in murine cardiac fibroblasts, as determined by qRT–PCR. **(f),** Effects of Mirt2 on the expression of inflammatory factors in human monocyte-derived macrophages, as determined by qRT–PCR. **(g),** Effects of Mirt2 on the expression of inflammatory factors in human hepatocytes, as determined by qRT–PCR. Data represent the mean ± SEM of three independent experiments. * $P < 0.05$ vs. Ad-EV group. Two-tailed Student's t-test for two groups.

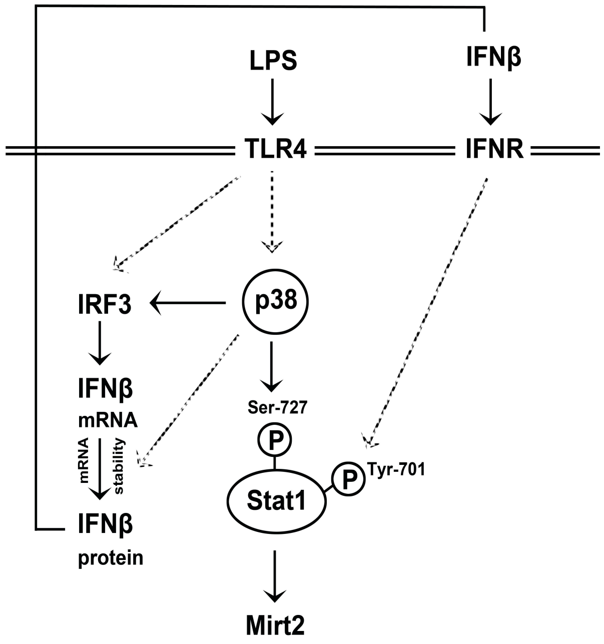

Supplementary Figure 6. The possible regulation mechanisms on Mirt2 expression.

Activation of LPS-p38-Stat1 (Ser-727) and LPS-IFN-α/β-Stat1 (Tyr-701) pathways transcriptionally promoted the expression of lncRNA Mirt2.

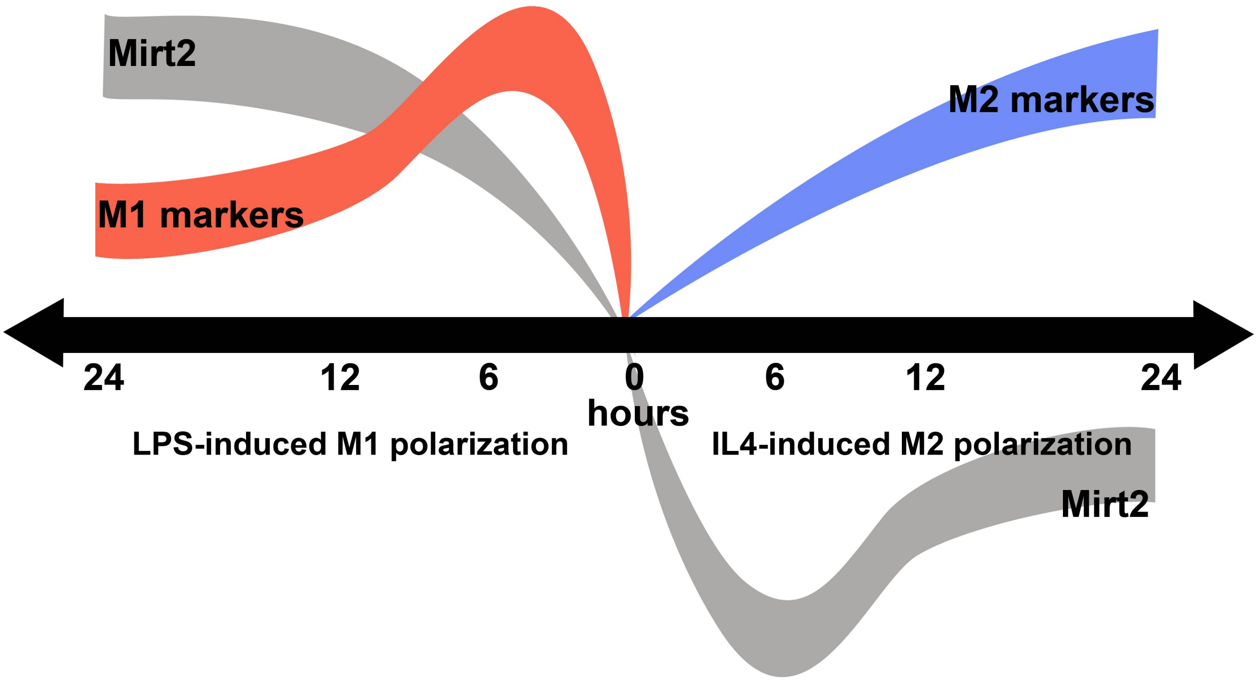

Supplementary Figure 7. The relative levels of Mirt2 and related markers of classical M1 and alternative M2 macrophages, induced by LPS and IL-4 respectively.

Figure 1d

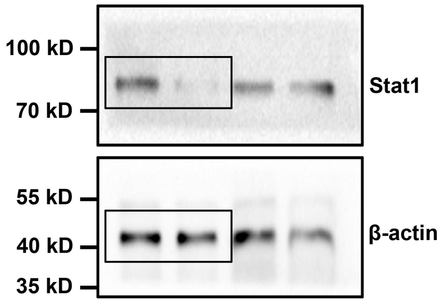

Figure 1k

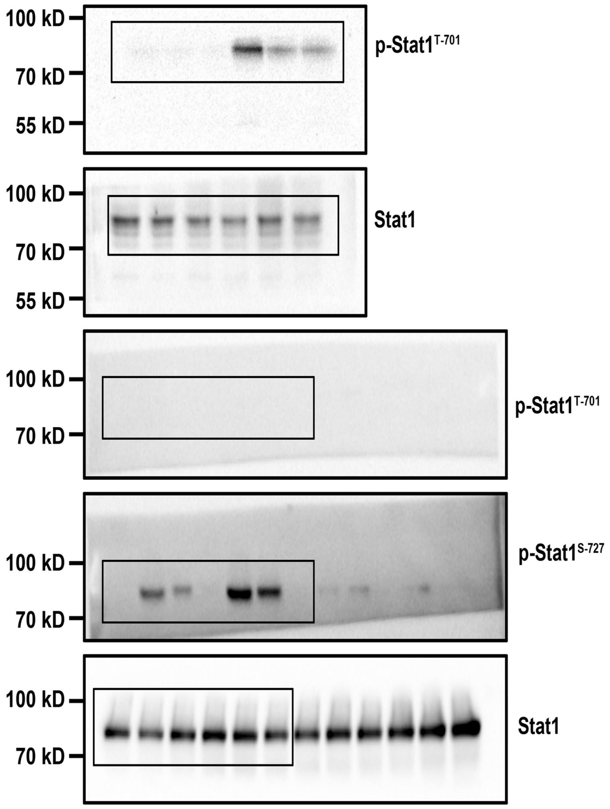

Figure 4g

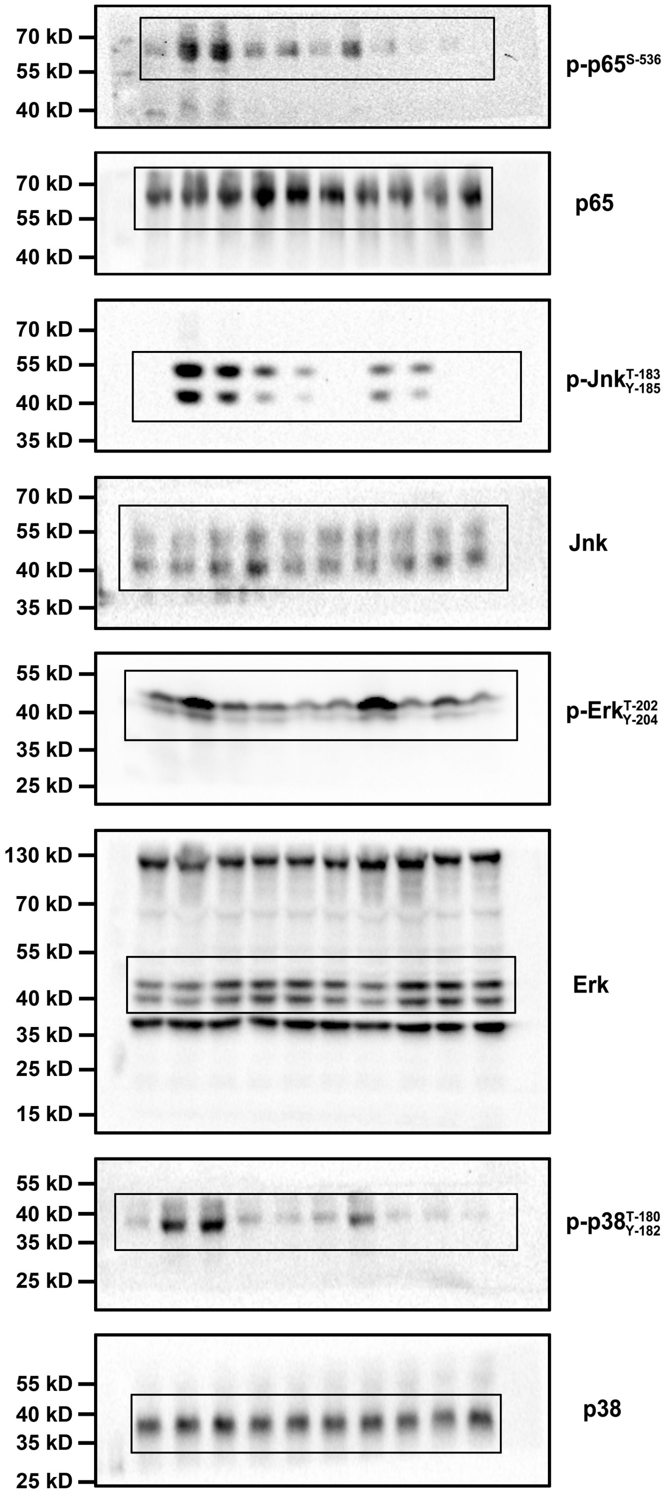

Figure 4g

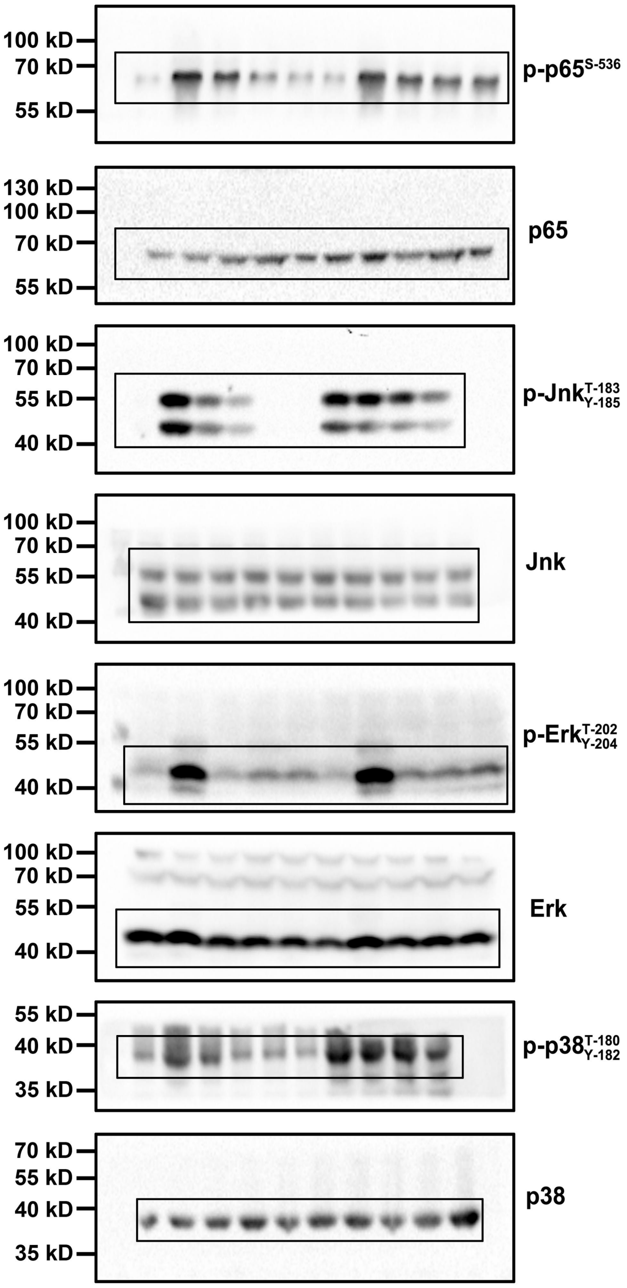

Figure 5a

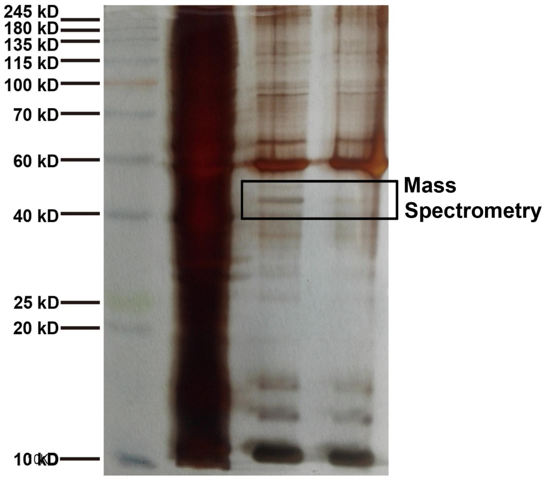

Figure 5b

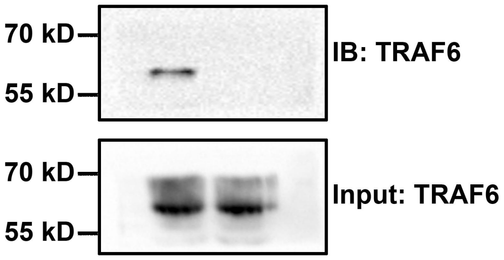

Figure 5e

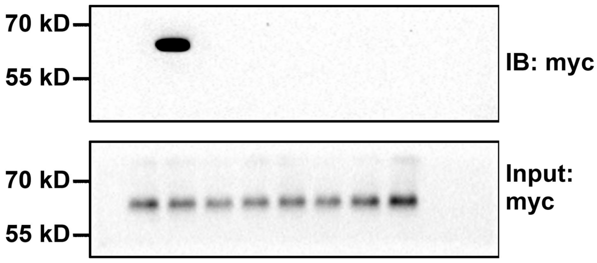

Figure 5f

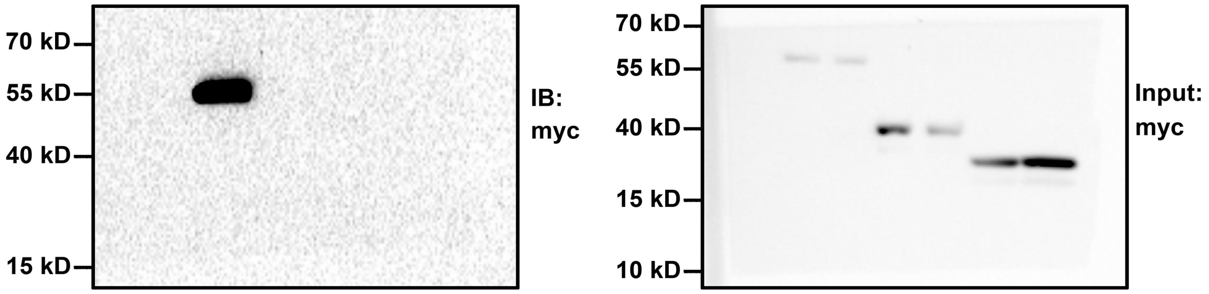

Figure 5f

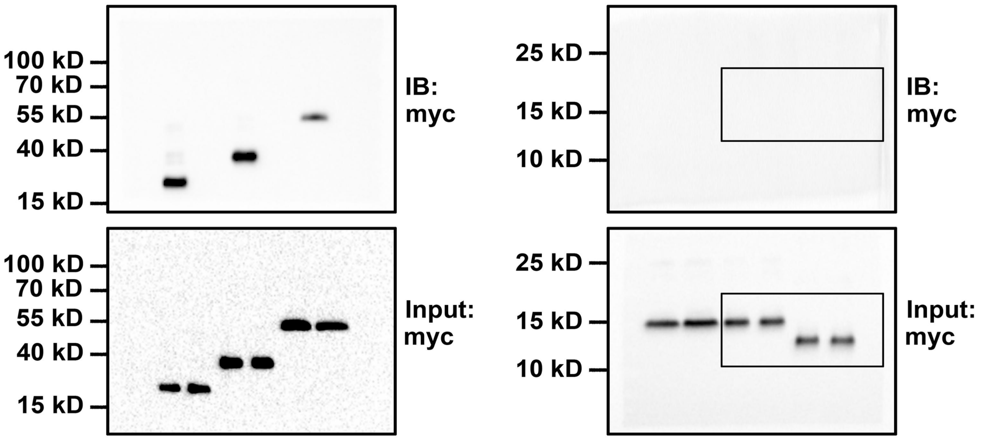

Figure 5i

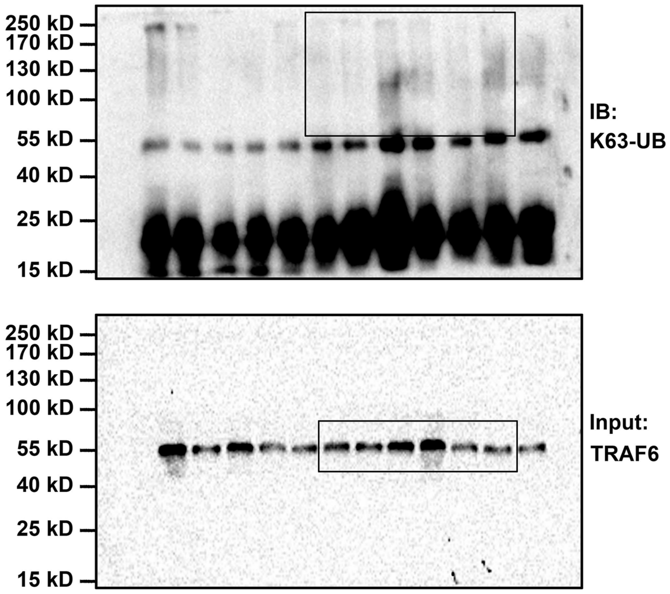

Figure 5g

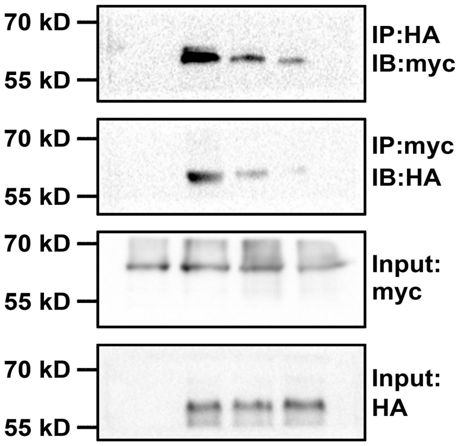

Figure 5h

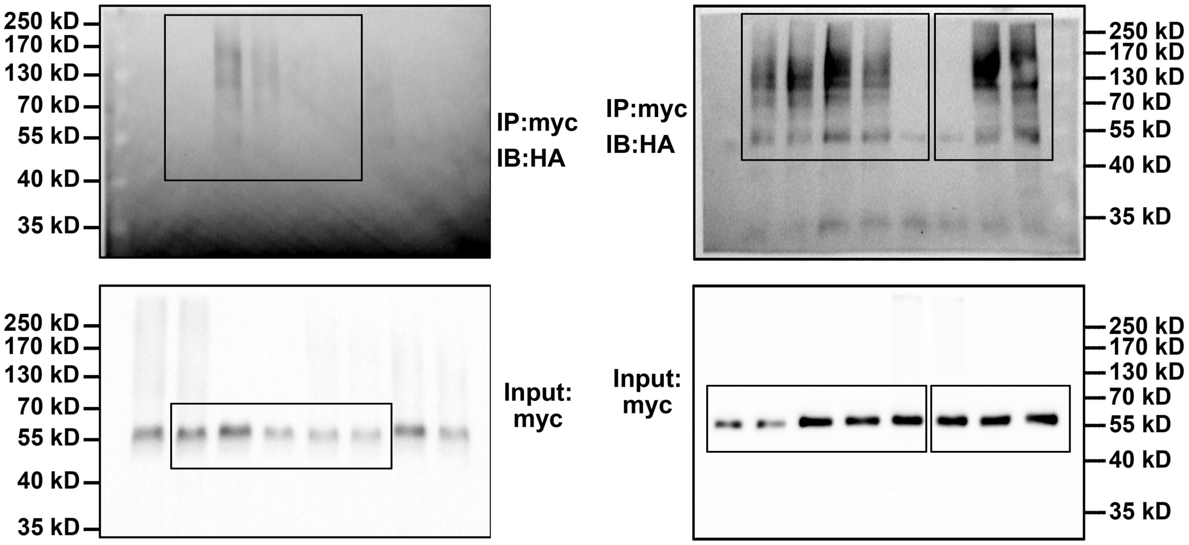

Figure 5j

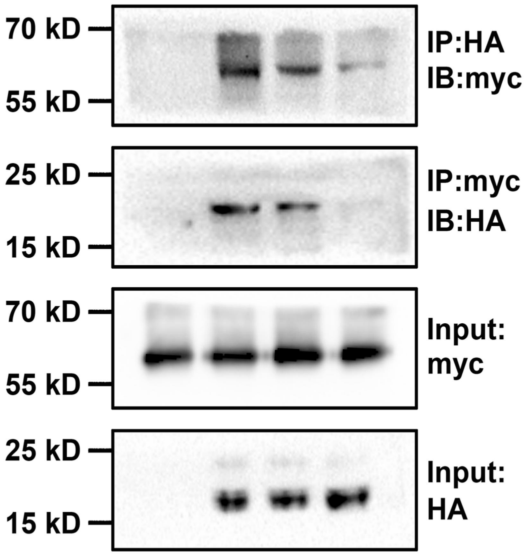

Figure 5k

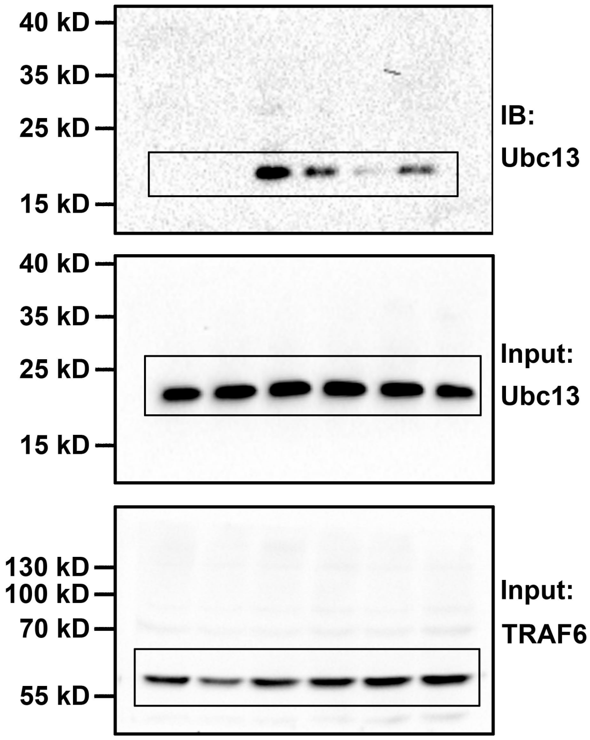

Figure 5l

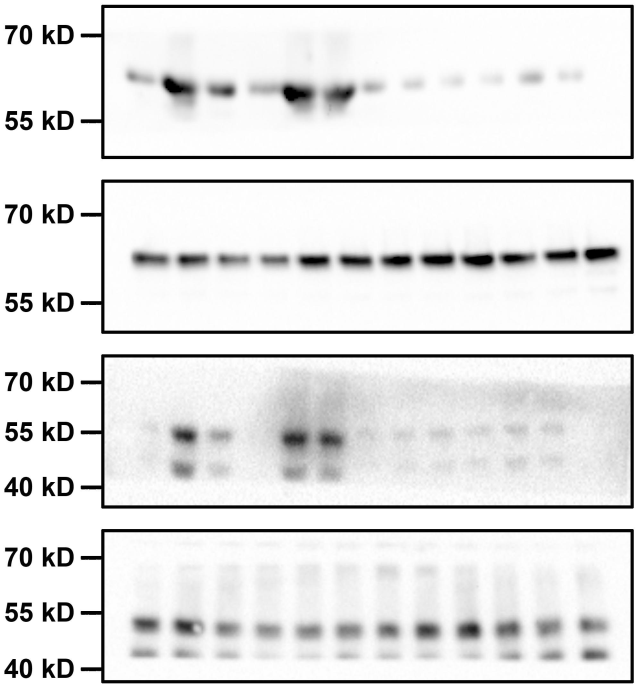

Figure 6f

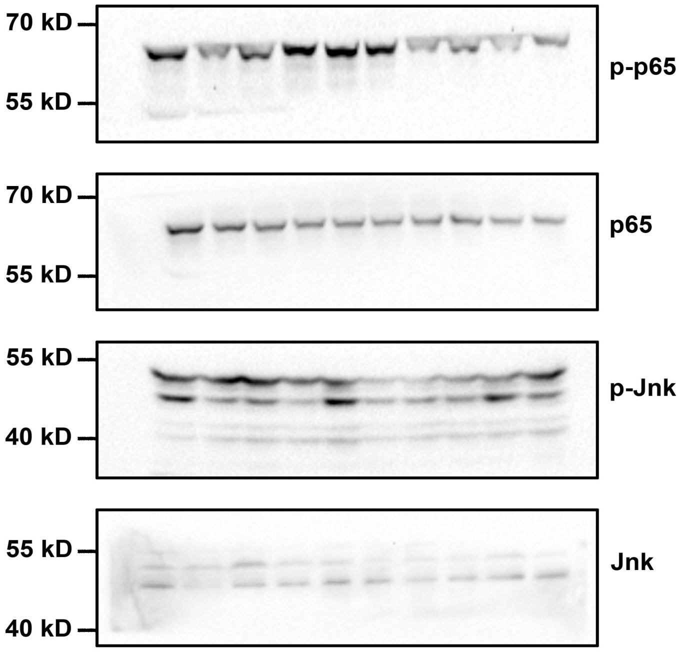

Figure 7b

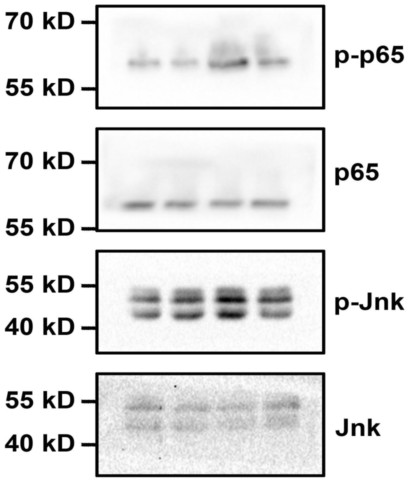

Figure 6j

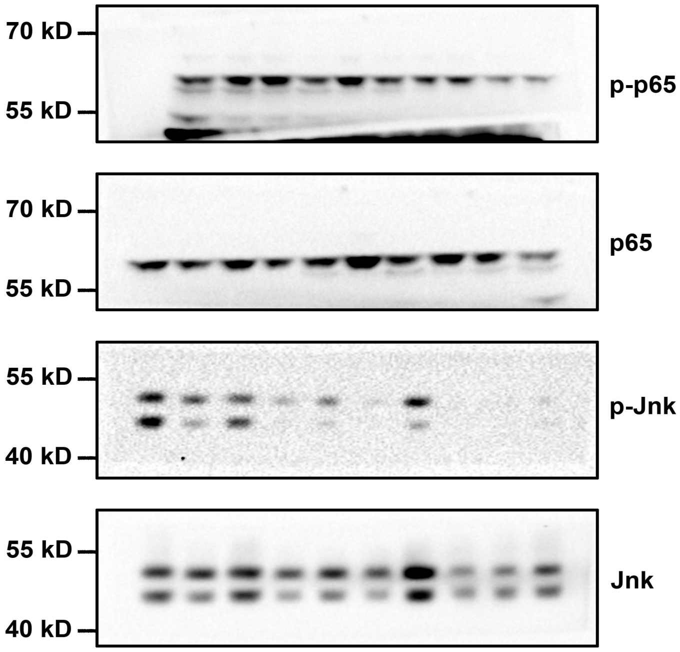

Figure 7d

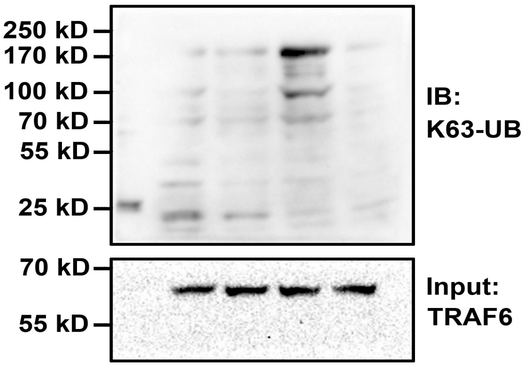

Supplementary Figure 8. Uncropped scans of western blots included in main figures.

Supplementary Table 1. qRT-PCR primers used

Gene	Forward primer	Reverse primer
Mirt2	TCAACACTTTCCATAGGT	ATTGTGAGGTCCAGATAG
TNF	CAGGCGGTGCCTATGTCTC	CGATCACCCCGAAGTTCAGTAG
IL-1β	GAAATGCCACCTTTTGACAGTG	TGGATGCTCTCATCAGGACAG
IL-6	CTGCAAGAGACTTCCATCCAG	AGTGGTATAGACAGGTCTGTTGG
IL-12	AGACATCACACGGGACCAAAC	CCAGGCAACTCTCGTTCTTGT
IFN-β	AGCTCCAAGAAAGGACGAACA	GCCCTGTAGGTGAGGTTGAT
isg15	GGTGTCCGTGACTAACTCCAT	TGGAAAGGGTAAGACCGTCCT
MCP1	TAAAAACCTGGATCGGAACCAAA	GCATTAGCTTCAGATTTACGGGT
IL-23α	CAGCAGCTCTCTCGGAATCTC	TGGATACGGGGCACATTATTTT
CXCL9	GGAGTTCGAGGAACCCTAGTG	GGGATTTGTAGTGGATCGTGC
CXCL10	CCAAGTGCTGCCGTCATTTTC	GGCTCGCAGGGATGATTTCAA
Arg1	CTCCAAGCCAAAGTCCTTAGAG	GGAGCTGTCATTAGGGACATCA
CD206	CTCTGTTCAGCTATTGGACGC	CGGAATTTCTGGGATTCAGCTTC
Mgl1	CAATGTGGTTAGTTGGATCGGC	CCCAGTTCTTAAAGCCTTTCTCA
Fizz1	CCAATCCAGCTAACTATCCCTCC	ACCCAGTAGCAGTCATCCCA
Ym1	CAGGTCTGGCAATTCTTCTGAA	GTCTTGCTCATGTGTGTAAGTGA
CCL22	CTCTGCCATCACGTTTAGTGAA	GACGGTTATCAAAACAACGCC
CCL24	ATTCTGTGACCATCCCCTCAT	TGTATGTGCCTCTGAACCCAC
CCL26	TTCTTCGATTTGGGTCTCCTTG	GTGCAGCTCTTGTCGGTGAA
